# Osteogenesis of bone marrow mesenchymal stem cell in hyperglycemia

**DOI:** 10.3389/fendo.2023.1150068

**Published:** 2023-06-21

**Authors:** Meng Luo, Zhihe Zhao, Jianru Yi

**Affiliations:** ^1^ State Key Laboratory of Oral Diseases & National Clinical Research Center for Oral Diseases, West China Hospital of Stomatology, Sichuan University, Chengdu, China; ^2^ Department of Orthodontics, West China Hospital of Stomatology, Sichuan University, Chengdu, China

**Keywords:** bone marrow mesenchymal stem cell, osteogenesis, hyperglycemia, diabetes mellitus, Reactive Oxygen Species

## Abstract

Diabetes mellitus (DM) has been shown to be a clinical risk factor for bone diseases including osteoporosis and fragility. Bone metabolism is a complicated process that requires coordinated differentiation and proliferation of bone marrow mesenchymal stem cells (BMSCs). Owing to the regenerative properties, BMSCs have laid a robust foundation for their clinical application in various diseases. However, mounting evidence indicates that the osteogenic capability of BMSCs is impaired under high glucose conditions, which is responsible for diabetic bone diseases and greatly reduces the therapeutic efficiency of BMSCs. With the rapidly increasing incidence of DM, a better understanding of the impacts of hyperglycemia on BMSCs osteogenesis and the underlying mechanisms is needed. In this review, we aim to summarize the current knowledge of the osteogenesis of BMSCs in hyperglycemia, the underlying mechanisms, and the strategies to rescue the impaired BMSCs osteogenesis.

## 1 Introduction

Diabetes mellitus (DM) is a metabolic disease characterized by hyperglycemia (1). Prolonged exposure to hyperglycemia results in a range of chronic complications, including cardiovascular diseases, chronic kidney diseases, nerve damage, and bone diseases, thereby greatly compromising individuals’ quality of life (2–5). The number of people suffering from DM was 537 million in 2021 and was projected to reach 643 million by 2030 (6). Currently, DM is one of the leading causes of death worldwide and has become a public concern of global health (7).

The skeletal complications caused by DM are well-documented. The skeletal fragility and risk of bone fractures increase in patients with DM (8, 9). Long-term exposure to hyperglycemia jeopardizes the healing of bone fractures (10). In addition, DM patients are predisposed to a higher risk of periodontitis and peri-implantitis (11–14). The molecular modulation of bone metabolism in high glucose (HG) conditions has been widely investigated. Massive evidence indicates that the osteogenic ability of bone marrow mesenchymal stem cells (BMSCs) is inhibited under HG conditions, which contributes to reduced bone turnover and impaired bone quality in DM patients (9). With the increasing incidence of DM and the resultant socioeconomic burden, it is of great significance to obtain a better understating regarding the impacts of the HG environment on BMSCs osteogenesis and the underlying mechanisms. Therefore, the aim of this review is to summarize and discuss the current knowledge about BMSCs osteogenesis under HG conditions and provide new insights into future research and clinical management of DM.

## 2 Osteogenesis of BMSCs

BMSCs are a type of multipotent progenitors in bone marrow, which can differentiate into osteogenic, adipogenic, myogenic, and chondrogenic lineages to support tissue homeostasis, repair, and regeneration (15, 16). The osteogenesis of BMSCs is vital for bone growth, fracture healing, and osseointegration (17, 18). To achieve osteoblastic function, BMSCs need to move from bone marrow niche to target tissues through blood circulation (19–22). The homing and migration of BMSCs is a complex process governed primarily by chemical factors including chemokines (23, 24), cytokines (25–27), and growth factors (28–33), as well as mechanical factors including mechanical strain (34, 35), shear stress (36), matrix stiffness (37), and microgravity (38).

Upon being recruited to the site where bone formation is required, the osteogenic commitment and differentiation of BMSCs which are delicately orchestrated by multiple intracellular signaling pathways and extracellular environment are of utmost importance to osteogenic activity (39). The early regulators of osteogenic commitment of BMSCs include Wnt/β-catenin signaling, bone morphogenetic proteins (BMPs), hedgehog proteins, and endocrine hormones (40, 41). After that, runt-related transcription factor 2 (Runx2) and Osterix 1 (Osx1) are crucial to shifting the gene expression of BMSCs to osteogenic genes that are responsible for type I collagen-based extracellular matrix deposition (42). Then, the BMSCs committed to osteogenic lineage gradually present the gene expression profile and morphological features of osteoblasts and later osteocytes, expressing osteoprotegerin (OPG), alkaline phosphatase (ALP), type I collagen, and osteocalcin (43). The osteoblasts and osteocytes can synthesize and secrete osteoid and mineralization factors to produce bone tissue, which couples with the osteoclastic activity, thus achieving bone modeling and remodeling to adapt to the metabolic and structural needs (44–46).

## 3 Impaired BMSCs osteogenesis under HG conditions

As stated, BMSCs osteogenesis, in a broad sense, is a complex process including migration, proliferation, osteogenic differentiation, etc. Plethora of research have suggested that HG conditions have comprehensive impacts on BMSCs osteogenesis. The most studied process implicated in the impaired BMSCs osteogenesis under HG conditions will be discussed in the following subsections, from the perspective of migration, proliferation, senescence, apoptosis, and osteogenic differentiation of BMSCs.

### 3.1 Migration of BMSCs

Although the mechanisms involved are incompletely understood, current evidence demonstrates the migration of BMSCs is mainly regulated by a series of cytokines and chemokines such as stromal cell-derived factor 1 (SDF-1) and CXC ligand 12 (CXCL12) (47, 48). Exposure to a hyperglycemic environment can alter the expression of related agents, thus modulating the migration of BMSCs. For example, HG conditions inhibited the migration and proliferation of BMSCs by reducing the expression of CXC receptor 4 (CXCR-4) *via* activating glycogen synthase kinase-3β (GSK3β) (49). Besides, the expression of integrin subunit alpha 10 (ITGA10) in BMSCs was down-regulated in the HG environment, which suppressed the migration and adhesion of BMSCs *via* FAK/PI3K/AKT/GSK3β/β-catenin pathway (50).

### 3.2 Proliferation of BMSCs

Proliferation of BMSCs is essential for bone growth and formation. However, the impaired proliferation and the consequent deficiency of BMSCs can significantly delay fracture healing and reduce the efficacy of regenerative therapy (51–54). The consensus on the inhibited BMSCs proliferation under HG conditions has been reached by previous studies. The decreased proliferative ability under HG conditions has also been reported in the MSCs derived from adipose tissue (55), gestational tissue (umbilical cord, placenta, and chorion) (56), and periodontal ligament tissue (57). Intriguingly, it has been reported that the HG treatment lower than 25 mM promoted the proliferation of BMSCs while the HG treatment of 35 mM displayed an inhibitory effect (58). The perplexing findings emphasize the significance to standardize the simulation of hyperglycemic settings *in vitro*. The glucose concentrations and HG treatment duration of the studies regarding BMSCs proliferation were summarized in **Table 1**, which showed substantial heterogeneity. Thus, it is of great significance to determine the appropriate HG treatment to diminish the heterogeneity of *in vitro* studies, and hopefully, provide more reliable evidence.

**Table 1:** Main information of the studies regarding BMSCs proliferation.

Authors, Year	Results	HG treatment
Xing Q et al., 2021 (59)	HG significantly decreased BMSCs proliferation compared with that of any of the other groups at each indicated time point.	25 mM for 24h, 48h and 72h
Zhang B et al., 2016 (49)	From days 3 to 7, proliferation was reduced in the presence of 16.5 mM compared with that at 5.5 mM.	16.5 mM for 7d
Gu Z et al., 2013 (60)	The ability of the BMSCs to proliferate was significantly reduced.	Not clear
Kim YS et al., 2013 (61)	In 7 days, the proliferation rate had significantly decreased and had been restored by oxytocin.	Higher than 250 mg/dl
Zhao YF et al., 2013 (62)	Proliferation of the diabetic BMSCs proceeded slower than the normal BMSCs at days 3, 5 and 7.	Higher than 16.7 mmol/l
Stolzing A et al., 2012 (63)	The formation of BMSC colonies was reduced.	25mmol/L for 2 weeks
Ezquer F et al., 2011 (64)	Although viable BMSCs were less abundant in diabetic bone marrow, they exhibited the same proliferation potential.	Higher than 400 mg/dl for more than 2 months
Jin, P et al., 2010 (65)	Proliferative abilities at day 3, 5 and 7 were lower.	Higher than 16.7 mmol/l
Stolzing A et al., 2010 (66)	While the sizes of the colonies were smaller, CFU numbers increased in 4-week diabetic rats but declined in 12-week ones.	Not clear
Gopalakrishnan V et al., 2006 (67)	Proliferation of BMSCs was decreased.	16.5 and 49.4 mmol/L for 3, 7, and 28 days

### 3.3 Senescence of BMSCs

Senescence is a cellular response featured with a stable cell cycle arrest that limits the potential of cell proliferation (68, 69). The senescent MSCs are characterized by decreased stemness, cell phenotype changes, immunomodulatory property damage, impaired proliferative ability, and higher susceptibility to apoptosis, which highly restricts their therapeutic value (70). Evidence have shown that hyperglycemic settings alter MSCs’ characteristics and functions, resulting in the senescence of MSCs (70, 71). The increased oxidative stress and reactive oxygen species (ROS) could be a main cause of senescence of BMSCs under HG conditions (72). BMSCs displayed a greater rate of senescence owing to the facilitated autophagy mediated by ROS in hyperglycemic environment (73). Mechanistically, HG induces ROS generation in BMSCs primarily through the activation of NADPH oxidase, while the generated ROS activates autophagy and upregulates the expression of aging markers (73). The relationship between TNF-α and stem cell senescence under HG conditions has also been noticed. TNF-α expression is elevated in obese and diabetic individuals (74, 75), while the addition of TNF-α significantly increased cellular senescence of stem cells (76). Therefore, inhibiting ROS or TNF-α production to relieve BMSCs senescence seems to be a plausible strategy for the treatment of bone complications induced by DM.

### 3.4 Apoptosis of BMSCs

Currently, most studies agree that the HG conditions promote the apoptosis of BMSCs. The molecular modulation of HG-induced apoptosis is complex. First, an excess of advanced glycation end products (AGEs) that refer to a family of compounds formed by non-enzymatic reactions between carbohydrates and proteins, lipids, or nucleic acids can be produced under HG conditions (77, 78). AGEs can bind to their multiple receptors (RAGEs) on BMSCs and elicit apoptosis *via* activating caspases through TNF-α production, p38 AMPK pathway, and oxidative stress (79). It has been shown that it is AGEs rather than HG conditions *per se* decrease the proliferation and increase the apoptosis of BMSCs *via* producing ROS and selective reduction of superoxide dismutase (SOD)-1 and SOD-3 (80). Besides, it should be noted that the up-regulation of RAGEs expression in BMSCs by HG conditions can amplify the pro-apoptotic effect of AGEs (81). Second, the increased ROS production and the resultant oxidative stress in BMSCs are prominent contributors to HG-induced apoptosis. The HG-induced ROS overproduction can be attributed to mitochondrial dysfunction (82–84) and the pathologic activation of the AGE-RAGE signaling pathway (79, 85). ROS overproduction results in high levels of free radicals, which further cause DNA damage and trigger apoptosis *via* activating the p53 pathway (86). In addition, the high levels of ROS under HG conditions enhance the expression of apoptotic proteins and inhibit anti-apoptotic proteins *via* regulating AKT signaling pathways, thus leading to apoptosis (87). Third, autophagy is involved in the HG-mediated apoptosis of BMSCs. Autophagy is a necessary process for the homeostasis and stemness of BMSCs. The activation of protective autophagy has been suggested to ameliorate hypoxia-induced pancreatic β cell apoptosis and senescence (88). Similarly, enhanced autophagy has been found to inhibit the apoptosis of BMSCs in a hyperglycemic setting (73). Moreover, the HG-induced apoptosis of BMSCs can be partially reversed by enhancing AMPK/mTOR pathway-mediated autophagy (89). Although the regulatory effects of autophagy on apoptosis of stem cells derived from other sources remain controversial (90–92), it is conceivable to reach a preliminary conclusion that enhanced autophagy protects BMSCs away from HG-induced apoptosis.

The reciprocal interaction between ROS, AGEs, and autophagy in the regulation of HG-induced BMSCs apoptosis have received increasing concern. It is believed that ROS acts as the master regulator. The ROS and oxidative stress which are induced and maintained by the HG environment result in the exacerbated formation of AGEs (93). The AGEs not only elicit the apoptosis of BMSCs but also further elevates the ROS levels, thus forming a vicious cycle and amplifying the apoptotic effect (79). In contrast, the excessive ROS in BSMCs activates autophagy through transcriptional and posttranslational mechanisms, whereas autophagy is thought to degrade impaired organelles and proteins to reduce the intracellular ROS (94, 95), thus keeping BMSCs away from the ROS-induced apoptosis under HG conditions. Overall, although the detailed molecular process remains enigmatic, the role of ROS, AGEs, and autophagy in HG-mediated BMSCs abnormality have been recognized. Future studies are still needed to decipher the detailed mechanisms.

### 3.5 Osteogenic differentiation of BMSCs

The most prominent feature of BMSCs is multipotency. BMSCs can differentiate into adipocytes and osteoblasts depending on the prevailing signaling molecules (16, 96). High glucose environments can induce alterations in the expression levels of various signaling molecules, which impact the osteogenic differentiation of BMSCs through signal transduction, gene expression, as well as immune regulation. The most widely studied mechanisms that underlie HG-mediated BMSCs osteogenesis include ROS overproduction (97, 98), AGEs-RAGEs signaling axis (99), and immunomodulation (100, 101). etc. Considering that the regulatory effects of ROS and AGEs have been discussed above, in this part we will focus on the signal transduction, gene expression, and immune regulation of BMSCs in hyperglycemia.

#### 3.5.1 Signal transduction

##### 3.5.1.1 Wnt pathways

Wnt signaling plays a central regulatory role in the osteogenic differentiation of BMSCs (102–105). HG conditions exert comprehensive disturbance to Wnt pathways, from the upstream regulator to the downstream core molecule. Wnt10b is critical to maintaining trabecular bone thickness and bone mineral density (106, 107), and the suppression of Wnt10b facilitated adipogenesis (108, 109). Whereas, the Wnt10b concentration in blood samples of DM patients was greatly lower than that of healthy individuals, and activating Wnt10b effectively reversed HG-induced osteogenic inhibition in BMSCs (110). Moreover, hyperglycemia has been reported to selectively enhance autogenous Wnt11 expression in BMSCs to stimulate adipogenesis through Wnt/protein kinase C pathway (109). Besides, HG conditions have been shown to activate GSK-3β, which compromised β-catenin stabilization and had negative effects on BMSCs osteogenesis (111, 112). Histone deacetylase 1 (HDAC1), a widely recognized inhibitor of β-catenin (113), has been found to be up-regulated under HG conditions, thus inhibiting the osteogenic differentiation of BMSCs (114). Additionally, hyperglycemia has been shown to increase sclerostin production, which induced adipogenesis of BMSCs by inhibiting Wnt signaling (115).

The regulation of miRNAs in BMSCs osteogenesis under HG conditions has been recognized in the past decade. It has been found that the HG-induced reduction of miR-124-3p expression greatly activated GSK-3β expression, thereby causing decreased osteogenesis in BMSCs (116). Furthermore, HG conditions upregulated the expression of miR-214-3p, which inhibited the osteogenesis of BMSCs by suppressing Wnt signaling *via* targeting β-catenin directly (117). Likewise, HG-induced upregulation of miR-493-5p inhibited ZEB2, thus preventing the nuclear accumulation of β-catenin and the subsequent osteogenesis of BMSCs (118). More basic research is needed to elucidate the mechanisms regarding the regulatory of HG conditions on miRNA expression and the precise roles of miRNAs in HG-induced inhibited BMSCs osteogenesis.

##### 3.5.1.2 PPARs pathways

Peroxisome proliferator-activated receptors (PPARs) are nuclear receptors that serve as transcription factors upon ligand activation and are implicated in numerous biological processes (119–121). Three known PPAR isotypes have been identified in mammals, termed PPARα, PPARβ/δ, and PPARγ (122).

The ties between PPARγ and Wnt signaling in BMSCs osteogenic and adipogenic differentiation have been widely investigated (123, 124). PPARγ is known to act as the master transcriptional regulator of adipogenic differentiation of BMSCs at the expense of repressed osteogenic differentiation (125, 126). It has been proven that the up-regulated expression of PPARγ in diabetic mice correlates with increased bone adiposity and impaired bone quality (127). Exposure to hyperglycemia has been shown to induce the adipogenic differentiation of BMSCs *via* the PI3K/AKT-regulated PPARγ pathway both *in vitro* and *in vivo* (128). In short, the increased PPARγ expression shifts BMSCs toward adipogenic lineage and reduces the osteogenic capability under HG conditions. The regulatory effects of PPARβ/δ on bone turnover were first recognized in 2013 (129). The activation of PPARβ/δ amplified Wnt-dependent and β-catenin-dependent osteogenesis, and the knockout of PPARβ/δ impaired bone remineralization and bone mass (129, 130). The involvement of PPARβ/δ in the BMSCs osteogenesis under HG conditions has been identified recently. PPARβ/δ has been found to be down-regulated in BMSCs under HG conditions, while the application of PPARβ/δ agonist rescued the osteogenic differentiation of BMSCs under HG conditions and promoted the bone regeneration of calvarial defects in diabetic rats by enhancing AMPK/mTOR pathway-mediated autophagy (89).

##### 3.5.1.3 PI3K/AKT pathways

Phosphoinositide 3-kinases (PI3Ks) are a family of intracellular phosphorylating enzymes involved in cellular functions including cell growth, apoptosis, senescence, and differentiation (131). The activation of PI3Ks mediates the phosphorylation of protein kinase B (AKT), a signal transducer, which regulates multiple downstream agents (132–136). GSK3β is a known downstream target of AKT phosphorylation and plays a pivotal role in bone formation and remodeling (137). It has been shown that the reduced Runx2 expression in BMSCs led to the inhibition of osteogenic differentiation by PI3K/AKT/GSK3β/β-catenin pathway under HG conditions (137, 138). Besides, the down-regulation of miR-32-5p induced by hyperglycemia has been found to inhibit BMSCs osteogenic differentiation through the PI3K/AKT/GSK3β pathway (139). Another study also identified that the phosphorylation of AKT and GSK3β reversed the inhibited osteogenic differentiation of BMSCs under HG conditions (140).

ROS has been found to regulate PI3K directly, thus modulating downstream signaling and the transcription of target genes (141). The elevated ROS levels under HG conditions have been shown to prevent the phosphorylation of AKT and the mechanistic target of rapamycin (mTOR), inducing osteogenic inhibition in BMSCs (142, 143). Moreover, the suppression of PI3K/AKT pathways caused the reduction of antioxidant factor Nuclear factor-erythroid factor 2-related factor 2 (Nrf2), which compromised ROS scavenging ability and aggravated oxidative stress, thereby forming a vicious cycle with osteogenic inhibition as the consequence (144). Besides ROS, it has been demonstrated that the reduction of periostin resulted in the osteogenic inhibition of BMSCs under HG conditions through the AKT pathway (145). Furthermore, the enhanced semaphorin3B expression has been demonstrated to alleviate the inhibition of osteogenic markers of BMSCs under HG conditions *via* PI3K/AKT pathways (59). Collectively, it has been well illustrated that PI3K/AKT/pathways are implicated in HG-mediated BMSCs osteogenesis and targeting PI3K/AKT/pathways might be a promising therapeutic approach to treat diabetic bone diseases.

##### 3.5.1.4 MAPK pathways

The mitogen-activated protein kinases (MAPKs) cascades encompass major signal transduction pathways that are involved in the regulation of cell morphology, growth, survival, differentiation, and apoptosis upon a wide range of stimuli including cytokines, growth factors, oxygen radicals, and cell-cell interactions (146–149). The conventional MAPKs are characterized into three groups, termed extracellular signal-regulated kinases (ERK),c-Jun amino (N)-terminal kinases (JNK), and p38 isoforms, with phosphatase-mediated cross-talk between these MAPK cascades (150). The regulation of ERK, JNK, and p38 pathways on human MSC osteogenesis has been identified in 2000 (151). Of the three pathways, ERK is the most extensively studied cascade and has been shown to be vital for BMSCs osteogenesis, while its actual impacts under HG conditions remain controversial. It has been reported that HG treatment suppressed the phosphorylation of ERK by increasing the expression of miR-221-3p and miR-222-3p, thus inhibiting the osteogenic differentiation of BMSCs (152). However, another study found that the phosphorylation of ERK was facilitated and MAPK/ERK pathway was activated in the impaired BMSCs osteogenesis under HG conditions (153). The different glucose concentrations might account for the perplexing findings, which need further investigation. The dephosphorylation of p38 and suppressed p38-MAPK pathway have also been found to correlate with the impaired BMSCs osteogenesis under HG conditions (154). The potential regulatory of JNK in this process is less well understood.

##### 3.5.1.5 BMP pathway

BMPs are members of the transforming growth factor superfamily, which play a crucial role in bone and cartilage formation (155). BMP-2 is critical in the osteoblastic differentiation of BMSCs (156, 157). However, the BMP-2 expression in BMSCs under HG condition was significantly decreased, which led to reduced expression of osteogenic markers and impaired osteogenesis of BMSCs (58). Smads are also pivotal for the BMP pathways, which transduce the signal to the nucleus and regulate the transcription of target genes (158). Under HG conditions, the increased miR-203-3p expression inhibited the osteogenesis of BMSCs *in vitro* and impaired jaw bone quality of diabetic rats *in vivo* by suppressing BMP/Smad pathway *via* targeting Smad1 (159, 160). Moreover, hyperglycemia has been reported to hinder BMSCs osteogenesis through inhibition of the BMP/Smad pathway by targeting Smad1, Smad4, and Smad5 (161).

#### 3.5.2 Epigenetic regulation

Epigenetic regulation, such as DNA methylation, histone acetylation, histone methylation and non-coding RNA, plays an important role in determining the differentiation direction of BMSCs (162, 163). Liu et al. discovered that diabetic rats with elevated levels of DNA methylation exhibited decreased bone mass and density. The *in vitro* application of 5-aza2’-deoxycytidine (5-aza-dC), a DNA methyltransferase inhibitor, to reduce the levels of DNA methylation, rescued the osteogenic differentiation capacity of MSCs under hyperglycemic conditions (164). MicroRNAs (miRNAs), a small noncoding RNA, play a key role in modulating various cellular life processes, and they regulate gene expression *via* targeting specific mRNAs (165). It was reported that miR-337 suppressed osteogenesis in high glucose-treated BMSCs by targeting the 3’-UTR region of Rap1A, and knockdown of miR-337 promoted osteogenic differentiation. These results suggest that upregulated miR-337 inhibits osteogenesis in high glucose (166). The level of miR-9-5p has also been found to be increased in HG condition. MiR-9-5p knockdown promotes HG-induced osteogenic differentiation BMSCs *in vitro* and mitigates the diabetic osteoporosis condition of rats *in vivo* by targeting DDX17 (167). Over-expression of miR-542-3p induced by HG inhibits the osteoblast differentiation, whereas inhibition of miR-542-3p function by anti-miR-542-3p promoted expression of osteoblast-specific genes, alkaline phosphatase activity and matrix mineralization (168). Therefore, epigenetics, as an important regulatory mechanism of BMSC differentiation, plays a crucial role in determining the direction of differentiation. However, the epigenetic regulation mechanism underlying osteogenic differentiation of BMSCs in high glucose environments remains insufficiently studied.

#### 3.5.3 Protein expression

Proteomics has been widely used as a powerful tool to investigate the protein expression on a large scale to identify potential biomarkers or activated proteins (169). One proteomic study found that high glucose levels can affect the expression of 12 proteins in BMSCs, including upregulation of annexin A7, fumarate hydratase, annexin A2, annexin A1 and alpha2-HS-glycoprotein. This may impair osteogenic differentiation and lead to glucose toxicity in diabetic conditions (170, 171). Additionally, tropomyosin alpha-1 chain was found to be downregulated in BMSCs cultured with high glucose, which plays roles in regulating cell proliferation, morphogenesis, vesicle trafficking and glucose metabolism. The results indicate that protein functions involved in bone formation can plausibly explain the bone deformability in patients with hyperglycemia (171–173). Although very little research has been done on proteomics, this is an area worth investigating as it could help us to better use stem cells in the field of tissue engineering.

#### 3.5.4 Macrophage immunomodulation

DM alters components of immune systems and has been regarded as an inflammatory disease (174). Macrophages are specialized innate immune cells that orchestrate the immune response, tissue repair, and inflammation (175, 176). Macrophages produce distinct functional phenotypes in response to specific stimuli and signals, which is termed macrophage polarization. The two polarization outcomes are the classically-activated M1 subtype and the alternatively-activated M2 subtype (177, 178). Both M1 and M2 macrophages are closely related to inflammatory responses. M1 macrophages secret pro-inflammatory agents including IL-1β, IL-6, and TNF-α, which inhibit osteogenic differentiation of BMSCs and have destructive effects on tissues (179–183). On the contrary, M2 macrophages exhibit an anti-inflammatory phenotype, which secrete multiple anti-inflammatory factors (184) and promote bone regeneration, and participate in tissue repair (182, 183). Studies have shown that the relationship between macrophages and BMSCs is reciprocal. As mentioned before, M2 macrophages can promote the osteogenic differentiation of BMSCs, while M1 macrophages have a negative effect. Meanwhile, BMSCs can significantly regulate the phenotype and function of macrophages (185–188). Hence, improving the inflammatory environment by modulating the polarization state of macrophages has been considered a potential approach for the treatment of related diseases (189).

In a hyperglycemic setting, the morphology of macrophages has been shown to adopt a fried egg-like shape with dense filopodia that presents a typical M1-like appearance, while the macrophages cultured in normal glucose condition showed a more typical round shape that suggests a resting state (101). Besides, the restoration of the electrical microenvironment has been found to enhance M2/M1 ratio, which further facilitated BMSCs osteogenesis and bone regeneration in diabetic conditions (101). Moreover, the hyperglycemia enhanced the macrophage infiltration and expression of pro-inflammatory factors by up-regulating CCL2, a key regulator for macrophage recruitment and polarization during inflammation (190), which ultimately inhibited osteogenic differentiation of BMSCs through MAPK pathways and facilitated alveolar bone loss (191).

## 4 Strategies to rescue BMSCs osteogenesis under HG conditions

The impaired osteogenesis of BMSCs under HG conditions is a complicated process with comprehensive mechanisms. The approaches facilitating the recruitment and osteogenic differentiation of BMSCs under HG conditions are in urgent demand for therapeutic purposes. Many attempts have been made to recover BMSCs osteogenesis in hyperglycemic settings to obtain sound clinical efficacy. Here, we summarized the approaches to attenuate the impaired BMSCs osteogenesis in the HG environment based on the aforementioned mechanisms.

The accumulation of ROS is a major cause of the impaired osteogenic capability of BMSCs. Hence, the methods targeting the removal of excessive ROS are promising to ameliorate impaired osteogenesis. Using the scaffolds with ROS-scavenging abilities greatly promoted the osteogenic differentiation of BMSCs and improved the efficiency of bone defect regeneration in diabetic rats (192, 193). Other antioxidants, such as high-density lipoprotein (194), chrysin (144), and silibinin (142), have also been reported to alleviate HG-suppressed osteogenesis of BMSCs *via* an antioxidant effect. Furthermore, as the lead candidate for treating diabetes, metformin has been identified to scavenge the overproduced ROS under HG conditions to rescue the inhibited osteogenesis of BMSCs by reactivating the AMPK/β-catenin pathway (54). However, it is noteworthy that the physiological amount of ROS concentration is vital for the proliferation and differentiation of BMSCs, thus the proper dosage of antioxidants in specific conditions is critical and remains to be prudently determined.

The potentials of AGEs and autophagy in recovering BMSCs osteogenesis under HG conditions have also been identified. The application of adrenomedullin 2 reversed the HG-impaired BMSCs *in vitro* and accelerated bone regeneration in diabetic rats *via* attenuating AGE-induced imbalances in macrophage polarization through PPARγ/NF-κB signaling (195). Morroniside treatment also recovered the osteogenic differentiation of BMSCs and reduced bone loss in diabetic rats by suppressing AGEs formation and RAGEs expression (196). In addition, the activation of PPARβ/δ improved the osteogenic differentiation of BMSCs under HG conditions and promoted the bone regeneration of calvarial defects in diabetic rats by enhancing AMPK/mTOR pathway-mediated autophagy (89). Similar to ROS, both AGEs formation and autophagy are physiological events that play a pivotal role in cell metabolism. Hence, their potential to result in unwanted effects such as DNA damage and tumorigenicity should not be ignored and deserve further investigation.

The alteration of the pro-inflammatory environment has been proposed to recover the HG-induced inhibition of BMSCs osteogenesis. It has been reported that a novel biomimetic electrical nanocomposite membrane could attenuate pro-inflammatory M1 macrophage polarization *via* PI3K-AKT signaling pathway, thereby enhancing the osteogenic differentiation of BMSCs and bone regeneration in rats with type 2 diabetes mellitus (101). The inhibition of inflammation using adrenomedullin 2 also exhibited similar effects in rats with type 1 diabetes mellitus (195). Moreover, the addition of BMP-4-loaded sustainable release nanoparticles into a scaffold reduced the levels of pro-inflammatory factors by promoting the polarization of RAW264.7 to M2 macrophages, which achieved favorable osteogenic activities both *in vitro* and *in vivo* on the basis of HG environment (100). Overall, the management of inflammation seems to be an ideal strategy allowing BMSCs to regain osteogenic ability under HG conditions, especially considering its benefits on other complications of DM such as kidney diseases, brain diseases, etc (197–199).

## 5 Conclusions

**Figure 1:**
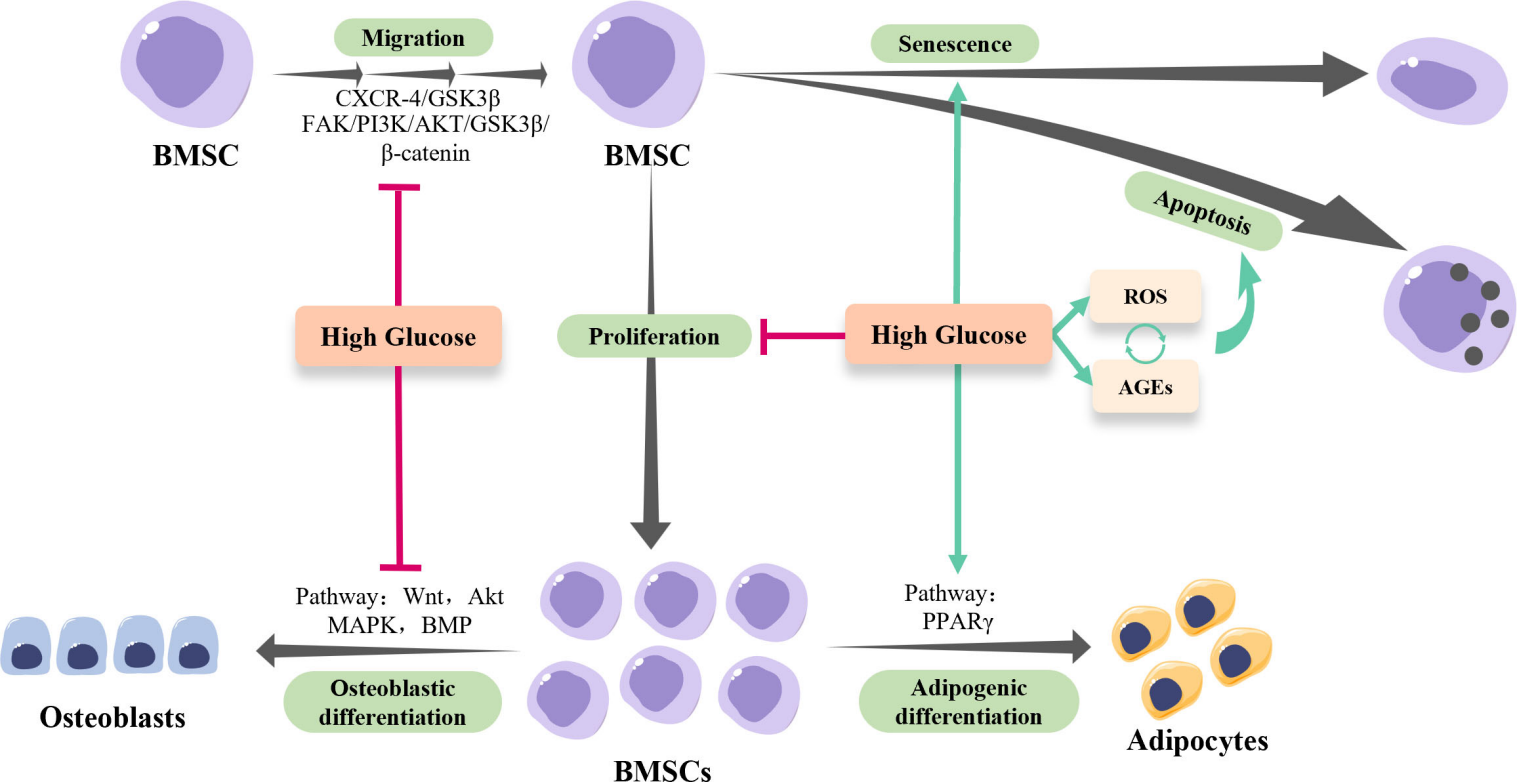
The osteogenesis of BMSCs in hyperglycemia. High-glucose conditions inhibit the migration and proliferation of BMSCs, while facilitate the senescence and apoptosis of BMSCs *via* regulating reactive oxygen species (ROS) and advanced glycation end products (AGEs) production. Of note, the excessive ROS generated by hyperglycemia results in the accumulation of AGEs, which in turn exacerbates ROS production, forming a positive feedback loop. In addition, high-glucose conditions shift BMSCs towards adipogenic lineage rather than osteogenic lineage *via* modulating multiple pathways.

Individuals living with DM are more vulnerable to skeletal complications. Massive evidence indicates that the impaired osteogenic ability of BMSCs in hyperglycemic settings is a main cause of diabetic osteopathy such as increased fracture risk and impaired bone healing (**Figure 1**). The osteogenesis of BMSCs is vital for bone growth and remodeling. In addition, BMSCs have been considered the ideal candidate for regenerative therapy to treat bone defects due to their easy accessibility, osteogenic potentials, and immunoregulatory function. Hence, revealing the mechanisms underlying and developing the strategies to rescue the HG-induced inhibition of BMSCs osteogenesis are of great significance for both diabetic bone diseases and regenerative therapy in diabetic patients.

The impacts of the HG environment on the osteogenic activities of BMSCs have been well characterized, while the underlying mechanisms are still elusive (**Figure 1**). Based on current evidence, it is conceivable to summarize that the HG conditions disable the injury site to recruit enough BMSCs by suppressing the migration and proliferation of BMSCs. Moreover, the HG conditions inhibit the osteogenic differentiation of BMSCs indirectly by promoting its senescence and apoptosis, and directly *via* multiple signaling pathways. As for the strategies, reducing intracellular ROS and suppressing AGEs-RAGEs axis activities seem to be effective, while the potential adverse effects should be studied in a holistic view. Modulating the pro-inflammatory micro-environment of DM might be more promising with relatively safe outcomes and fewer adverse effects.

In short, we have summarized the detailed impacts of HG conditions on the osteogenesis of BMSCs and the underlying mechanisms, the approaches to rescue the diminished osteogenesis of BMSCs and facilitate the BMSCs-based regenerative medicine in the diabetic environment and proposed some suggestions for future research.

## Author contributions

ZZ and JY jointly conceived the manuscript. ML and JY performed the literature research and writing of the manuscript. ZZ revised the manuscript. All authors contributed to the article and approved the submitted version.
